# Targeting Adenosine Receptors: A Potential Pharmacological Avenue for Acute and Chronic Pain

**DOI:** 10.3390/ijms21228710

**Published:** 2020-11-18

**Authors:** Fabrizio Vincenzi, Silvia Pasquini, Pier Andrea Borea, Katia Varani

**Affiliations:** 1 Department of Translational Medicine and for Romagna, Pharmacology Section, University of Ferrara, 44121 Ferrara, Italy; psqslv@unife.it (S.P.); vrk@unife.it (K.V.); 2 University of Ferrara, 44121 Ferrara, Italy; bpa@unife.it

**Keywords:** adenosine, pain, adenosine receptors, antinociception

## Abstract

Adenosine is a purine nucleoside, responsible for the regulation of multiple physiological and pathological cellular and tissue functions by activation of four G protein-coupled receptors (GPCR), namely A_1_, A_2A_, A_2B_, and A_3_ adenosine receptors (ARs). In recent years, extensive progress has been made to elucidate the role of adenosine in pain regulation. Most of the antinociceptive effects of adenosine are dependent upon A_1_AR activation located at peripheral, spinal, and supraspinal sites. The role of A_2A_AR and A_2B_AR is more controversial since their activation has both pro- and anti-nociceptive effects. A_3_AR agonists are emerging as promising candidates for neuropathic pain. Although their therapeutic potential has been demonstrated in diverse preclinical studies, no AR ligands have so far reached the market. To date, novel pharmacological approaches such as adenosine regulating agents and allosteric modulators have been proposed to improve efficacy and limit side effects enhancing the effect of endogenous adenosine. This review aims to provide an overview of the therapeutic potential of ligands interacting with ARs and the adenosinergic system for the treatment of acute and chronic pain.

## 1. Introduction

Today, although substantial progress has been made, many pathological pain conditions remain poorly understood and resist currently available treatments. There is, therefore, a need for novel molecular targets to develop new therapeutic agents with improved efficacy and tolerability. Many experimental reports have identified adenosine receptors (ARs) as potential targets for the management of acute and chronic pain.

**Figure 1:**
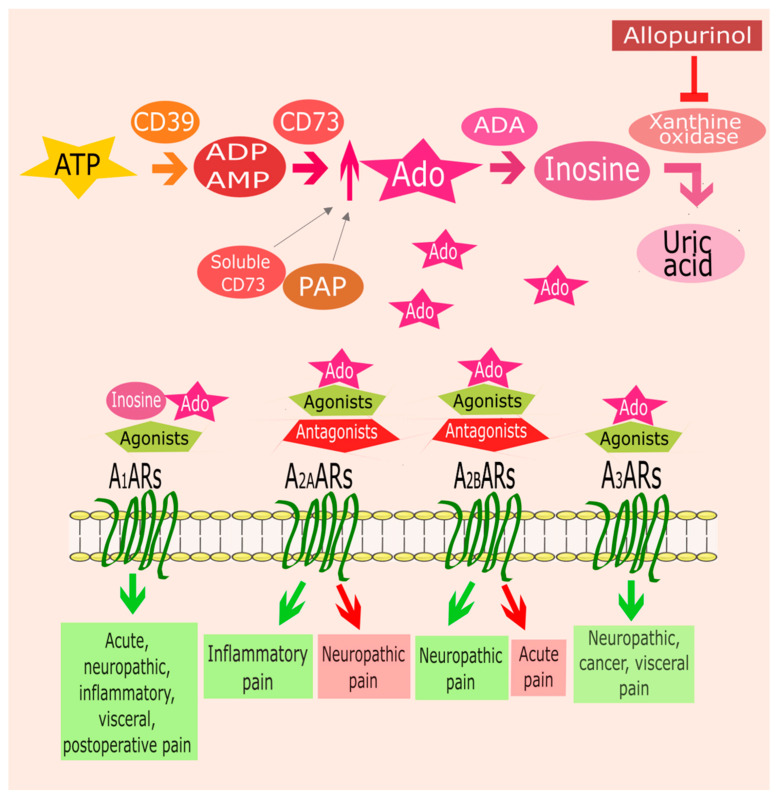
Adenosine (Ado) metabolism and involvement of adenosine receptors (ARs) in pain. The main source of adenosine is adenosine triphosphate (ATP) released from various cell types in response to different stimuli. ATP is dephosphorylated to adenosine diphosphate (ADP)/adenosine monophosphate (AMP) and then to adenosine by two ectonucleotidases (CD39, CD73). In nociception, the elevated levels of adenosine may alter the pain signaling. Thus, the modulation of adenosine metabolisms, increasing its levels, could represent an alternative strategy for pain management. Soluble CD73 provokes long-lasting thermal antihyperalgesic and mechanical antiallodynic effects through A_1_AR activation. Prostatic acid phosphatase (PAP), acting as an ectonucleotidase, induces A_1_AR-dependent antinociceptive effects in inflammatory and neuropathic pain models. Extracellular adenosine is rapidly metabolized to inosine by adenosine deaminase (ADA). Inosine is able to bind A_1_ARs, with an affinity similar to that of adenosine, inducing antinociceptive effects. Another strategy to promote the accumulation of inosine is represented by the inhibitors of the enzyme xanthine oxidase such as allopurinol. In the extracellular space, adenosine can interact with its receptors. A_1_ARs stimulation with adenosine, adenosine metabolites like inosine, or synthetic agonists presents analgesic effects in acute, neuropathic, visceral, postoperative, and inflammatory pain. Activation of A_2A_ARs by endogenous adenosine or exogenous agonists results in antinociception in case of inflammatory pain. While, A_2A_ARs blockade shows analgesic effects in neuropathic pain. Regarding A_2B_ARs, their stimulation has antinociceptive effects in neuropathic pain and their blockade is useful for acute pain treatment. Finally, A_3_ARs activation gives analgesic effects in different types of pain such as neuropathic, cancer, and visceral pain.

Adenosine is a ubiquitous endogenous autacoid that mediates its physiopathological effects by interacting with four G protein-coupled receptors (GPCR), namely A_1_, A_2A_, A_2B_, and A_3_ ARs [1]. A_1_ and A_3_AR are coupled with G_i_ and G_o_ members of the G protein family, through which they have an inhibitory effect on adenylyl cyclase (AC) activity, while A_2A_ARs and A_2B_ARs stimulate it by coupling to G_s_ proteins. The consequent modulation of cyclic adenosine monophosphate (cAMP) levels activates or inhibits a large variety of signaling pathways depending on the specific type of cell involved. Although there are instances in which adenosine exerts detrimental effects in various pathological conditions, it is generally considered a protective and homeostatic mediator against tissue damages and stress conditions [2,3]. In physiological and unstressed conditions, the extracellular concentrations of adenosine are maintained low as a result of the rapid metabolism and uptake [4]. However, its levels rise considerably during conditions involving increased metabolic demand, hypoxia, inflammation, and tissue injury. In particular, increased levels of extracellular adenosine were observed in pathological conditions such as epilepsy [5,6], ischemia [7,8], cancer [9,10], inflammation [11], and ultimately pain [12,13]. Although adenosine can be produced intracellularly, the main source of adenosine in pathological states is adenosine triphosphate (ATP), released by cells under stressful conditions and dephosphorylated from the combined action of two hydrolyzing enzymes termed ectonucleoside triphosphate diphosphohydrolase (CD39) and ecto-5′-nucleotidase (CD73) [1]. Regarding nociception, these elevated levels of endogenous adenosine can alter pain transmission by actions at spinal, supraspinal, and peripheral sites. The extracellular action of adenosine can then be terminated by its transformation to inosine through adenosine deaminase (ADA) and/or by intracellular uptake via nucleoside transporters [14]. Intracellularly, adenosine is phosphorylated to AMP by adenosine kinase or deaminated to inosine by ADA. Given these regulation mechanisms of adenosine concentration, potential pain management can be obtained not only with specific ligand interacting with ARs but also by manipulating endogenous tissue levels of adenosine by modulating its metabolism or transport [13] (Figure 1). 

Although adenosine and its receptors represent a clear target for pharmacological treatment of various diseases and pathological states including pain, very few drugs acting on the adenosinergic system have so far reached the market. The reason behind this discrepancy may be partly due to the ubiquitous distribution of ARs in almost every cell and tissue, making it difficult to avoid unwanted side effects. In recent years, many efforts have been made to improve our understanding of the role of adenosine in nociception and identify novel strategies to exploit the therapeutic potential of the adenosinergic system such as selective ligands, partial agonists, allosteric modulators, or adenosine concentration modulating agents. 

The focus of the present review is to describe the recent advances in our understanding of the role of ARs in nociception. For each receptor subtype, we will briefly summarize and discuss the preclinical experimental studies that investigated their role and mechanism of action in the modulation of acute and chronic pain.

## 2. ARs and Pain

### 2.1. A1ARs

The antinociceptive effect of adenosine has been primarily attributed to the activation of A_1_ARs [15] and various A_1_AR agonists or positive allosteric modulators have been shown to be effective in several preclinical models of pain (Table 1). The signaling pathway underlying A_1_ARs antinociception includes inhibition of cyclic AMP and consequently protein kinase A (PKA) activation, inhibition of Ca^2+^ channels, activation of K^+^ currents, and interactions with phospholipase C (PLC), inositol triphosphate (IP3), diacylglycerol (DAG), extracellular signal-regulated kinases (ERK), and β-arrestin pathways [3]. The prominent role of this receptor subtype in analgesic responses is due to its peculiar expression in different sites relevant to pain transmission. A_1_ARs are indeed located on peripheral sensory nerve endings in the spinal cord dorsal horn, and at supraspinal pain-processing structures [13,16]. Microglia represent another important localization for the antinociceptive action of A_1_ARs, especially for pain states involving glial activation [17]. The peripheral activation of A_1_ARs diminished inflammatory hypernociception caused by carrageenan intraplantar administration. Using specific inhibitors, the antinociceptive effect of the A_1_AR agonist CPA was shown to be dependent on the nitric oxide (NO)/cyclic guanosine monophosphate (cGMP)/protein kinase G (PKG)/K_ATP_ signaling pathway [18]. The contribution of peripheral A_1_ARs to antinociception was further corroborated when the selective A_1_AR antagonist DPCPX reversed the antinociceptive effects of locally and systemically administered acetaminophen or tramadol in the formalin test [19]. A proof of the supraspinal antinociceptive action of A_1_AR has been reported in a study where the A_1_AR agonist 2′-Me-CCPA injected into the intra-periaqueductal grey (PAG) reduced pain behavior in the plantar and formalin tests. When microinjected into the PAG, 2′-Me-CCPA decreased the ongoing activity of the pronociceptive ON cells and increased the ongoing activity of the antinociceptive OFF cell in the rostral ventromedial medulla [20]. In neuropathic pain rats, the A_1_AR agonist CPA reduced thermal and mechanical sensitivity, while in naïve rats it decreased hypersensitivity to heat but not to mechanical stimuli. In this study, electrophysiological experiments suggested that spinal application of CPA depressed long-term potentiation of A- and C-fiber evoked field potentials while it depressed the baseline of C-fiber but not A-fiber response. To explain this different response, authors have hypothesized that A_1_ARs may be more expressed at C-fiber nerve endings than at A-fiber endings. [21]. In resiniferatoxin-induced neuropathy, the downregulation of A_1_ARs was suggested to contribute to nociception, while the intrathecal injection of adenosine attenuated mechanical allodynia, an effect abrogated by A_1_AR antagonism [22]. 

A_1_ARs also seem to be involved in visceral antinociception. Centrally injected agonist CPA increased the threshold volume of colonic distension-induced abdominal withdrawal reflex in conscious rats. Besides, the use of the A_1_ antagonist DPCPX suggested that adenosinergic signaling via A_1_ARs is also involved in the central orexin-induced antinociceptive action against colonic distension [23]. In a subsequent study, the authors suggested that serotonin 5-HT_1A_, 5-HT_2A_, dopamine D1 or cannabinoid CB_1_ receptors, and the opioid system might specifically mediate the CPA-induced visceral antinociception [24].

The potential role of A_1_ARs in postoperative pain was also investigated. Intrathecal administration of the A_1_AR agonist R-PIA decreased nonevoked spontaneous pain behavior and increased withdrawal thresholds after plantar incision. The opening of K_ATP_ channels contributed to this antinociceptive effect [25]. In a mouse model of acute postoperative pain, ankle joint mobilization decreased hyperalgesia through the involvement of peripheral and central A_1_ARs [26]. In another report, intrathecal adenosine injection inhibited hyperalgesia in two neuropathic pain models but not in a postoperative pain model represented by the plantar incision. However, in this model A_1_AR mRNA and protein expression were decreased suggesting that the lack of antinociceptive effect of adenosine on postoperative pain was due to the decrease in A_1_ARs [27]. 

An intriguing connection has been uncovered between A_1_ARs and acupuncture, an invasive practice worldwide used to relieve pain. Many studies report that the antinociceptive effects of acupuncture are dependent upon A_1_AR activation. It was shown that extracellular adenosine concentration is increased during acupuncture in mice and A_1_AR expression is required for the adenosine-mediated analgesic effect of acupuncture [28]. The involvement of A_1_ARs in the reduction in neuropathic pain exerted by electroacupuncture was demonstrated by the intrathecal injection of the A_1_AR antagonist DPCPX in a chronic constriction injury (CCI) model. In this report, the effect of A_1_ARs was related to the inhibition of astrocyte activation. [29]. Similar results were obtained in a Complete Freund’s adjuvant (CFA)-induced inflammatory pain mouse model, corroborating the involvement of A_1_ARs in electroacupuncture-mediated antinociception [30]. In another study, the analgesic effect of electroacupuncture was suggested to be mediated by overexpressed A_1_ARs in the spinal cord [31].

Different studies suggested that A_1_AR activation is required for the antinociceptive action of various natural compounds. Indeed, the A_1_AR antagonist DPXPC blocked the effect of norisoboldine, a benzylisoquinoline alkaloid isolated from *Radix Linderae* that diminishes pain response, in the formalin and writhing test [32]. In addition, A_1_AR is necessary to the analgesic effect of paeoniflorin, the major active component extracted from *Paeonia lactiflora*. In a study carried out in mice, paeoniflorin increased the mechanical threshold and prolonged the thermal latency after partial sciatic nerve ligation (SCNL), an effect abolished by the A_1_AR antagonist CPT or the genetic deletion of A_1_ARs [33]. In the hot plate test, the antinociceptive effect of (–)-linalool, a natural occurring enantiomer in essential oils, was blocked by both an A_1_ and an A_2A_AR antagonist [34]. D-Fructose-1,6-bisphosphate is an intermediate in the glycolytic pathway, inhibiting hyperalgesia induced by intraplantar injection of carrageenin and its mechanism of action seems dependent on adenosine accumulation that in turns exerts antinociceptive effects by activating peripheral A_1_ARs [35].

Adenosine is rapidly metabolized to inosine by ADA. Interestingly, different studies have identified inosine as a putative endogenous ligand of A_1_ARs and demonstrated the A_1_-mediated antinociceptive effect of the more stable metabolite of adenosine. In particular, inosine binds to A_1_ARs with an affinity resembling that of adenosine and induces antinociceptive, antiallodynic, and antihyperalgesic effects. In rats, both the A_1_AR antagonist DPCPX and the A_2A_AR antagonist ZM241385 reversed the antiallodynic, and antihyperalgesic effects of inosine in models of mechanical and heat hyperalgesia induced by bradykinin and phorbol 12-myristate 13-acetate [36]. In the formalin test, inosine did not induce antinociception in A_1_ARs knockout (KO) mice and the A_1_AR antagonist DPCPX inhibited its effects [37]. In a subsequent study, DPCPX, but not the A_2A_AR antagonist SCH58261, abrogated the antinociceptive effect of inosine in the intraplantar glutamate test [38]. A different strategy to promote the accumulation of purines like adenosine or inosine is by using the xanthine oxidase inhibitor, allopurinol. Indeed, it has been reported that intraperitoneal administration of allopurinol increased cerebrospinal fluid concentrations of adenosine and its metabolites inducing antinociceptive effects in different pain models. The selective A_1_AR antagonist DPCPX, but not the selective A_2A_AR antagonist SCH58261, prevented allopurinol-induced anti-nociception [39,40]. Since extracellular adenosine is primarily derived from the hydrolysis of AMP, the antinociceptive effect of a soluble version of the recombinant CD73, the enzyme that converts AMP to adenosine, has been tested in different pain models. The results of this study revealed long-lasting thermal antihyperalgesic and mechanical antiallodynic effects that were dependent on A_1_AR activation [41]. Prostatic acid phosphatase (PAP) acts as an ectonucleotidase hydrolyzing extracellular AMP to adenosine in nociceptive dorsal root ganglia neurons [42,43]. Intrathecal injection of a secretory version of human PAP induced A_1_AR-dependent antinociceptive effects in inflammatory and neuropathic pain models [44,45]. Furthermore, the injection of PAP into the popliteal fossa—a common acupuncture point—reduces pain responses in mouse models that lasted up to six days after a single injection, an effect dependent upon A_1_AR activation [46].

Several papers in the literature proposed a link between opioid-mediated antinociception and A_1_ARs. In a rat with spinal cord injury (SCI), it was demonstrated a supra-additive interaction between the adenosine A_1_AR agonist R-PIA and morphine in the reduction in mechanical allodynia-like behavior [47]. In spinal cord neuronal nociceptive responses, the antinociceptive effects of the A_1_AR agonist CPA were associated with activation of κ-opioid receptors since the reversal of the CPA effect was observed with norbinaltorphimine (a selective κ-opioid receptor antagonist) but not with low doses of µ-opioid antagonist naloxone [48]. While the opioid antagonist naltrexone did not affect the antinociception induced by CPA in the formalin test, the activation of A_1_ or A_2A_AR counteracted the µ-opioid receptor increase induced by formalin in the spinal cord, confirming the interaction between adenosinergic and opioid systems [49]. In a rat model of nerve ligation injury, the intrathecal administration of morphine synergistically enhanced the antiallodynic effect of the A_1_AR agonist R-PIA, suggesting an interaction between µ-opioid receptors and A_1_ARs at the spinal level [50]. In addition, other works reported that the antiallodynic/antihyperalgesic effect of morphine is reversed in the presence of the selective A_1_AR antagonist DPCPX [51] or in A_1_ARs KO mice [52]. Beyond opioids, the involvement of A_1_ARs has been observed in the antinociceptive effect of non-steroidal anti-inflammatory drugs such as acetaminophen. In the formalin test, when acetaminophen was administered systemically or locally, its antinociceptive effects were reversed by the intraplantar injection of the A_1_AR antagonist DPCPX, suggesting a link between activation of peripheral A_1_ARs and acetaminophen effects [53]. The contribution of spinal A_1_ARs to the action of acetaminophen secondarily to the involvement of descending serotonin pathways and the release of adenosine within the spinal cord was also suggested [54]. The involvement of A_1_ARs was also demonstrated in the antinociceptive effects of amitriptyline [55,56], oxcarbazepine [57], levetiracetam [58], and neuropeptide S [59].

Collectively, these preclinical studies provide strong support for the therapeutic potential of A_1_AR agonists. However, limited clinical efficacy and relevant cardiovascular and central adverse effects have, to date, hampered the development of A_1_AR agonists as analgesic drugs. An alternative approach to increase selectivity and reduce the possibility of adverse effects exploiting the physiological action of endogenous adenosine is the development of A_1_AR-positive allosteric modulators [60,61]. These agents enhance the function of receptors activated by endogenous agonists, they are expected to have a much lower side effect potential than an exogenous orthosteric ligand, a low propensity for receptor desensitization, and a high selectivity for a given receptor subtype [62]. T62 was the first A_1_AR-positive allosteric modulator to be tested in animal models of pain. Intrathecal or systemic administration of T62 reduced mechanical hypersensitivity induced by spinal nerve ligation (SNL) [63,64], reversed thermal hypersensitivity in carrageenin-inflamed rats [65], and was effective for postoperative hypersensitivity following plantar incision [66]. More recently, TRR469 was characterized as one of the most potent A_1_AR-positive allosteric modulators so far synthesized being able to increase adenosine affinity by 33 fold [67,68,69]. TRR469 effectively inhibited nociceptive behaviors in the formalin and writhing tests, with effects comparable to morphine. Furthermore, it revealed an antiallodynic action in the streptozotocin (STZ)-induced diabetic neuropathic pain model without inducing locomotor or cataleptic side effects as the orthosteric-acting CCPA did [69].

**Table 1:** A_1_AR ligands with antinociceptive effects in preclinical models of pain.

Ligand	Pharmacological Behavior	Pain Model	Species	Route of Administration
2′-Me-CCPA	agonist	formalin test	rat	intra-PAG, i.p. [20]
		plantar test	rat	intra-PAG, i.p. [20]
		tail flick test	rat	intra-PAG, i.p. [20]
CCPA	agonist	formalin test	mouse	i.p. [69]
		writhing test	mouse	i.p. [69]
		STZ-induced mechanical allodynia	mouse	i.p. [69]
		CFA induced-mechanical allodynia and thermal hyperalgesia	mouse	Zusanli acupoint-injection [28]
		SCNL induced-mechanical allodynia and thermal hyperalgesia	mouse	Zusanli acupoint-injection [28]
CPA	agonist	formalin test	mouse	i.p. [49]; i.t. [54]
		CFA-induced-mechanical allodynia and thermal hyperalgesia	mouse	i.m. [30]; i.p. [42]; Weizhong acupoint-injection [46]
		carrageenan-induced mechanical allodynia	rat	i.pl. [18]
		PGE_2_-induced mechanical allodynia	rat	i.pl. [18]
		SCNL-induced mechanical allodynia and thermal hyperalgesia	rat	i.p. [21]
		colonic distension-induced visceral pain	rat	s.c., i.c. [23,24]
R-PIA	agonist	plantar incision-induced mechanical allodynia	rat	i.t. [25]
		photochemical SCI-induced mechanical and thermal allodynia	rat	i.t. [47]
		SCNL-induced mechanical allodynia	rat	i.t. [50]
		photochemical sciatic nerve injury-induced mechanical and thermal allodynia	rat, mouse	i.t. [52]
		carrageenan-induced mechanical and thermal allodynia	rat, mouse	i.t. [52]
T62	positive allosteric modulator	SNL-induced mechanical allodynia	rat	i.p. [63]; i.t. [63,64]; p.o. [64]
		carrageenan-induced thermal hyperalgesia	rat	i.t. [65]
		plantar incision-induced mechanical allodynia	rat	i.t. [66]
TRR469	positive allosteric modulator	formalin test	mouse	i.p. [69]
		writhing test	mouse	i.p. [69]
		STZ-induced mechanical allodynia	mouse	i.p. [69]

PAG (periaqueductal grey); i.p. (intraperitoneal); STZ (streptozotocin); CFA (Complete Freund’s adjuvant); SCNL (sciatic nerve ligation); i.t. (intrathecal); i.m. (intramuscular); i.pl. (intraplantar); s.c. (subcutaneous); i.c. (intracisternal); SCI (spinal cord injury); SNL (spinal nerve ligation); p.o. (per os).

### 2.2. A_2A_ARs and Pain

The presence of A_2A_AR_S_ both on neurons and on glial cells is at the basis of A_2A_ARs implications in pain [70]. The relation between A_2A_ARs and pain has been controversial with evidence sustaining either pronociceptive and antinociceptive activity depending on the receptors’ localization and the kind of pain (Table 2) [13]. Studies supporting the pronociceptive role of A_2A_AR report that the selective blockade of this receptor subtype by systemic administration of SCH58261, a selective A_2A_AR antagonist, is able to counteract nociception; even the administration at the spinal level produced an equal effect [13,54]. These results are supported by experimental models of acute and nerve injury pain in A_2A_ARs KO which showed a decreased algesic reaction to pain tests and even a reduction in markers of neural activity [71]. Moreover, the administration of caffeine, which is a well-known non-selective antagonist of ADA, avert the sleep deprivation due to hypersensitivity following surgical operation. A_2A_AR selective blockade with ZM241385 has shown to decrease surgical pain levels and the thermal hyperalgesia caused by sleep deprivation in rats. These results support the hypothesis that A_2A_ARs are implicated in the regulation of the interplay between sleep and pain [72]. The pronociceptive effect of A_2A_AR stimulation was further corroborated in a study reporting that carrageenan-induced hyperalgesia was significantly reduced in A_2A_AR KO mice compared to wild type controls. Interestingly, the A_2A_AR inverse agonist ZM241385 injected into the hindpaw reduced the nociceptive behavior following carrageenan in female wild type mice, but not in males suggesting a sex difference in response to A_2A_AR activation in the periphery [73]. In addition, a series of inverse agonists showing two different affinity values for the A_2A_ARs with the high affinity value in the picomolar/femtomolar range was recently synthesized [74,75] and tested for their antinociceptive properties. In particular, one of these potent inverse agonists, namely TP455, proved to be more potent than morphine in writhing and tail immersion tests in mice [74].

Furthermore, the blockade of A_2A_ARs could provide protection in cases of neuropathic pain, which is one of the most common kinds of chronic pain, and it is found in different disorders and could lead to nerve dysfunctionalities [76]. Neuropathic pain pathophysiology is extremely intricate because it comprises central and peripheral mechanisms such as changes in ion channel expression, neurotransmitter release, and pain pathways [77]. Even oxidative stress could play an important role in the neuropathic pain origin process [78]. A body of evidence reveals that, after SCI, there are events that trigger reactive oxygen species (ROS) formation pathways such as microglia activation and glutamate release [79,80]. The injury at the sensory nerves level also involves damage to nuclear and mitochondrial DNA, and loss of antioxidant enzymes [81,82,83]. In fact, numerous studies report that the anti-oxidant or ROS scavengers administration has analgesic effects in many in vivo models of neuropathic pain. Furthermore, neuropathic pain is often a consequence of antitumoral treatments containing platinum because these drugs can provoke peripheral neuropathy and chemotherapy-induced oxidative stress is one of the important pathogenic factors damaging peripheral sensory neurons [84]. Recently, it has been proved that novel A_2A_AR antagonists featuring antioxidant moieties can reduce pain associated with oxaliplatin treatment in a mouse model of neuropathy reducing ROS level [85,86]. After peripheral nerve injury, A_2A_ARs stimulation induces both activation and proliferation of microglia and astrocytes responsible for inflammation occurring in neuropathic pain, while genetic deletion of the A_2A_ARs decreases all the behavioral and histological signs of pain [77,87]. Several studies also showed that systemic and spinal administration of the selective A_2A_AR antagonist SCH58261 has antinociceptive effects in different preclinical models [54,74].

Notwithstanding the coherence of the studies testifying for a pronociceptive role of A_2A_ARs, in the literature there is evidence even for an antinociceptive role. In particular, since A_2A_ARs are expressed in immune cells where they exert a potent anti-inflammatory action, their stimulation may be helpful in cases of inflammatory pain [3,13]. A_2A_ARs KO animals under prolonged inflammatory conditions show an up-regulation of markers of spinal cord neural activation. In these KO mice, the loss of the antinociceptive A_2A_ARs on immune cells exceeds the decrease in pronociceptive A_2A_ARs on nerve terminals leading to enhanced pain signaling [88]. It is well known that the stimulation of A_2A_ARs has anti-inflammatory effects but less is known about A_2A_AR agonists treatment and chronic inflammatory pain. Different studies report that A_2A_ARs expression is up-regulated in lymphocytes of rheumatoid arthritis patients, these data should represent a basis for further investigations in this field [89,90]. The selective agonist of A_2A_AR CGS21680 shows the ability to slow down disease progression in an in vivo model of arthritis [91]. Even in a rat animal model, it has been demonstrated that CGS21680 treatment was very effective in decreasing clinical features in comparison to standard antirheumatic drugs such as methotrexate and etanercept [92]. The treatment with the A_2A_AR agonist CGS21680 was also able to inhibit the nuclear factor kappa-light-chain-enhancer of activated B cells (NF-κB) activation and to reduce the release of inflammatory cytokines such as tumor necrosis factor-α (TNF-α), IL-1β, and IL-6. Besides, the A_2A_AR stimulation leads to a decrease in metalloproteinases 1 and 3 [93]. Finally, in another mice model of monoarthritis, a new A_2A_AR agonist, named LASSBio-1359, showed an important analgesic effect in response to inflammatory pain. This treatment was also able to reduce inflammation by decreasing TNF-α, inducible NO synthase (iNOS) expression, and joint damage [94]. The results of the above-mentioned studies highlight a role for A_2A_AR agonists as a potential therapeutic tool in the management of inflammatory pain [89,93]. 

Different reports demonstrated an antinociceptive role of A_2A_AR activation in models of neuropathic pain. An acute administration of A_2A_AR agonists, such as ATL313 and CGS21680, leads to an analgesic effect that lasts for many weeks and reverses the mechanical allodynia and thermic hyperalgesia while decreasing the markers of microglia and astrocytes activation [95]. Interestingly, the effect of A_2A_ARs activation was just specific for nerve injury or sensitized state suggesting a potential role of A_2A_AR agonists for neuropathic pain. Moreover, the blockade of A_2A_ARs by using a receptor antagonist in the presence of an anti-IL-10 antibody reverted the effect of ATL313, suggesting that the observed effects were due to the activation of A_2A_ARs and the simultaneous enhanced IL-10 production [95]. In a subsequent study, ATL313 induced long-lasting protection against allodynia caused by CCI, SNL, and sciatic inflammatory neuropathy (SIN), through a mechanism involving PKA and protein kinase C (PKC) [96]. In a recent study, a single intrathecal injection of the A_2A_AR agonists CGS21680 reversed mechanical allodynia in a rat model of SCI termed spinal neuropathic avulsion pain for at least 6weeks [97]. In the follow-up work, the peri-sciatic injection of the agonist ATL313 also demonstrated the efficacy of A_2A_AR activation at the site of nerve injury. These anti-allodynic effects were accompanied by a reduction in interleukin (IL)-1β and NO release, and reduced expression of iNOS and sciatic markers of monocytes/macrophages [98]. These studies revealed that the agonism toward A_2A_ARs was able to reduce different kinds of neuropathic pain such as inflammatory neuropathic pain and traumatic ones. In all these cases the A_2A_ARs stimulation averted and reverted the nociceptive stimuli amplification [98]. Additionally, the long time span of the analgesic effect after a single treatment suggests that A_2A_AR agonists could be useful for central neuropathic pain therapy. It is worth noting that in these studies, the antiallodynic effects of A_2A_AR agonists were associated with diminished reactive gliosis. Glial cells have a pivotal role in starting and carrying on neuropathic pain, and for this reason, many studies are directed toward the discovery of new strategies in order to defeat the pain expansion directed by glia. In recent years, A_2A_AR agonists have emerged as possible candidates for glial inhibition thanks to their capability to suppress inflammation in immune cells; consequently, A_2A_AR agonists represent a promising tool for the treatment of chronic pain of neuroinflammatory origin [99]. 

The activation of the A_2A_AR subtype also seems to be involved in the analgesic effect of neuropeptide S observed in the formalin test. Intracerebroventricular administration of this eicosapeptide reduced formalin-induced nociception during both phases 1 and phase 2 of the test, an effect counteracted by the non-selective AR antagonist caffeine or the selective A_2A_AR antagonist ZM241385 [59]. Besides, an interaction between A_2A_ARs and the opioid system was reported when the antinociceptive effect exerted by the intracerebroventricular injection of Adonis, an agonist-like monoclonal antibody with high specificity for the A_2A_ARs, was counteracted by naloxone, a non-selective opioid antagonist [100].

**Table 2:** A_2A_AR ligands with antinociceptive effects in preclinical models of pain.

Ligand	Pharmacological Behavior	Pain Model	Species	Route of Administration
ATL313	agonist	CCI-induced mechanical allodynia and thermal hyperalgesia	rat	i.t. [95,96]; peri-sciatic nerve injection [98]
		SNL-induced mechanical allodynia	rat	i.t. [96]
		SIN-induced mechanical allodynia	rat	i.t. [96]
		SCI-induced mechanical and thermal allodynia	rat	i.t. [97]
CGS21680	agonist	formalin test (early phase)	mouse	i.p. [49]
		CFA-induced-mechanical allodynia and thermal hyperalgesia	rat	i.p. [92]
		CCI-induced mechanical allodynia and thermal hyperalgesia	rat	i.t. [95,96]
		SCI-induced mechanical and thermal allodynia	rat	i.t. [97]
LASSBio-1359	agonist	formalin test	mouse	i.p. [94]
		carrageenan induced-mechanical allodynia and thermal hyperalgesia	mouse	i.p. [94]
Adonis	agonist-like monoclonal antibody	hot plate test	mouse	i.c.v. [100]
		tail flick test	mouse	i.c.v. [100]
TP455	inverse agonist	writhing test	mouse	i.p. [74]
		tail immersion test	mouse	i.p. [74]
ZM241385	antagonist	writhing test	mouse	i.p. [74]
		tail immersion test	mouse	i.p. [74]
		carrageenan induced-mechanical allodynia	mouse	s.c. [73]
		sleep deprivation-induced thermal hyperalgesia	rat	i.c.v. [72]
		plantar incision-induced mechanical allodynia and thermal hyperalgesia	rat	i.c.v. [72]

CCI (chronic constriction injury); i.t. (intrathecal); SNL (spinal nerve ligation); SIN (sciatic inflammatory neuropathy); SCI (spinal cord injury); i.p. (intraperitoneal); CFA (Complete Freund’s adjuvant); i.c.v. (intracerebroventricular); s.c. (subcutaneous).

### 2.3. A_2B_ARs and Pain

A_2B_ARs are expressed both at the central level and in the periphery: among pain-relevant sites, they are localized on immune-inflammatory cells, where they have pro-inflammatory functions, in the spinal cord, and on astrocytes [1,101,102]. Since adenosine presents a lower affinity for A_2B_ARs in comparison to other AR subtypes, A_2B_ARs are more involved when adenosine concentration rises, for example in pathological conditions such as hypoxia/ischemia and inflammation [2,103]. 

Nonetheless, the different functions of A_2B_ARs in various tissues and their involvement in the pathogenesis of pain are poorly known. As a consequence, more studies are needed in order to clarify their pro or anti-nociceptive actions in different types of pain conditions [11,101]. 

Unfortunately, studies on the relationship between pain and A_2B_ARs are limited due to the lack of selective ligands (Table 3). One of the first studies using selective A_2B_AR antagonists reported an antinociceptive activity of A_2B_ARs blockade in an acute pain model represented by the hot plate test. One of these ligands, PSB-1115, did not penetrate the blood brain barrier due to its polar sulfonate group, suggesting that peripheral A_2B_ARs were implicated in the analgesic activity [104]. Interestingly, the efficacy of morphine was enhanced by subeffective doses of these A_2B_AR antagonists. In a follow-up study, the systemic administration of PSB-1115 decreased the algesic response and edema in both phases of the formalin test [105]. In the same test, the selective blockade of A_2B_ARs by using alloxazine resulted in a dose-dependent reduction in nociceptive behavior [106]. Moreover, it has been reported that the treatment with A_2B_AR antagonists, MRS1754 and PSB-1115, was able to decrease pain in visceral hypersensitivity rat models [107,108]. PSB-1115 also reverted the antinociceptive effect of diphenyl diselenide, organoselenium compounds, in the hot plate test in mice [109].

A_2B_ARs seem to be involved even in chronic pain, with evidence highlighting that these receptor subtypes stimulate the interactions between immune cells and neurons. It was reported that high extracellular adenosine levels activate A_2B_ARs on myeloid cells, and that this leads to the activation of pain sensory neurons giving rise to hypersensitivity and chronic pain [110]. Intriguingly, the author demonstrated that A_2B_AR stimulation caused nociceptor hyperexcitability and promoted chronic pain via soluble IL-6 receptor trans-signaling. From these results, it is possible to deduce that the blockade of A_2B_ARs may repress the nociceptive activity.

All these findings seem to testify for a pronociceptive role of A_2B_ARs. However, it has been reported that even the activation of A_2B_ARs, using a selective agonist (BAY606583), presented an analgesic effect in an accredited model of neuropathic pain, in a similar way to A_2A_AR agonists treatment [96]. As it is well known, both A_2A_ARs and A_2B_ARs lead to increased cAMP accumulation and activation of downstream pathways; they also probably have a similar spinal mechanism of action. Normally, A_2B_AR stimulation activates PKA and the pathway of PLC/IP3/DAG leading to changes in gene transcription, while β-arrestins are responsible for the receptor internalization mechanism [13,111].

**Table 3:** A_2B_AR ligands with antinociceptive effects in preclinical models of pain.

Ligand	Pharmacological Behavior	Pain Model	Species	Route of Administration
BAY606583	agonist	CCI-induced mechanical allodynia	mouse	i.t. [96]
PSB-10	antagonist	formalin test	mouse	i.p. [105]
PSB-36	antagonist	formalin test	mouse	i.p. [105]
PSB-1115	antagonist	formalin test	mouse	i.p. [105]

CCI (chronic constriction injury); i.t. (intrathecal); i.p. (intraperitoneal).

### 2.4. A_3_ARs and Pain

A_3_ARs are present at the peripheral level in many tissues including inflammatory cells; they are less expressed in the central nervous system, nonetheless their activation causes functional effects, in particular, in glial cells [112]. The possibility to exploit A_3_AR stimulation, using selective agonists, has been studied in different pathologies counting cancer and inflammation [112,113].

A_3_ARs involvement has also been investigated in relation to pain; the first pieces of evidence reported a pronociceptive role [114]. Further studies, using more selective ligands, overturned previous results showing that A_3_AR agonists present antinociceptive activity so, they can be useful as analgesics especially for neuropathic pain (Table 4) [113]. In fact, the systemic administration of selective A_3_AR agonists, such as IB-MECA, Cl-IB-MECA and MRS1898, reduced the mechanical allodynia in a model of neuropathic pain—especially IB-MECA was as efficacious as morphine. The specificity of this effect was demonstrated by blocking A_3_ARs with the selective antagonist MRS1523, which abrogated the analgesic effect of A_3_AR agonists [115]. Interestingly, the A_3_AR agonists have no effects in acute pain models, for instance, hot plate and tail flick tests [116]. Another A_3_AR selective agonist, named MRS5698, was demonstrated to be able to reduce mechanical allodynia in different models of neuropathic pain. MRS5698 had an analgesic effect in acute pain tests but its activity persisted with repeated administrations [117]. The mechanism of action of this agonist involves GABA signaling: the A_3_ARs activation normalizes the changes in GABA concentrations caused by nerve damages, thus restoring the GABA inhibitory effect on pain transmission [118]. Moreover, it has been noticed that A_3_ARs stimulation inhibits N-type calcium channel opening in isolated rat dorsal root ganglion neurons, causing a reduction in the neurotransmitter release and the neuronal excitation [119]. In another model of nerve injury that produces tactile allodynia, the daily administration of IB-MECA averted the appearance of hypersensitivities, the activation of glial cells and the altered transmission of nociceptive stimuli, resulting in an attenuation of neuropathic pain [120]. In a recent study, MRS7476, a prodrug with increased aqueous solubility compared with parent MRS5698, was found to be efficacious in reversing neuropathic pain induced by CCI [121].

Anticancer chemotherapeutic treatments often induce neuropathy as an adverse effect; the stimulation of A_3_ARs can help to decrease the pain in these cases. The A_3_AR agonist IB-MECA is able to reduce the allodynia and the hyperalgesia induced by different anticancer drugs such as paclitaxel, oxaliplatin and bortezomib without diminishing their antitumoral effectiveness; even other A_3_AR agonists, Cl-IB-MECA and MRS1898 present the same effects [115,116]. The pathway involved seems to imply NF-κB, ERK and p38 inhibition and the production of inflammatory cytokines. In particular, the treatment with A_3_AR agonists reduces the release of the pro-inflammatory cytokines TNF-α and IL-1β while increases the anti-inflammatory IL-10 [122]. Other mechanisms have been proposed to explain the antinociceptive activity of A_3_ARs; among these are the diminished activation of astrocytes, inhibition of cAMP, PKA, the interaction with the PLC/IP3/DAG and phosphoinositide 3-kinase (PI3K)/mitogen-activated protein kinase (MAPK)/ERK/cAMP response element-binding protein (CREB) pathways and the signaling via Gi [123]. Even in the A_3_AR subtype, the internalization of the receptor is mediated by β-arrestins [111]. Recently, in a model of cancer chemotherapy-induced neuropathic pain, the A_3_AR agonist MRS5698 attenuated pro-inflammatory IL-1β production and promoted anti-inflammatory and neuroprotective IL-10 expression by regulating the nucleotide-binding oligomerization domain-like receptor protein 3 inflammasome [124].

Besides the antinociceptive effect of A_3_AR agonists in cancer pain and neuropathic pain related to chemotherapy, they have also found to be potent antitumoral agents in many animal models of different forms of cancer (melanoma, prostate, colon, and hepatocellular carcinoma), where they are able to reduce tumor growth [113,125]. Their therapeutic potential has also been assessed in a model of bone cancer pain in which mammary gland tumoral cells were injected into the tibia [126]. In this model, the repeated administration of Cl-IB-MECA decreased tumor growth, mitigated the nociception and the bone degradation associated with cancer. In addition, the A_3_AR agonist was also effective in delaying the onset and the advancement of bone cancer with a major efficacy when the treatment with Cl-IB-MECA was done before the injection of cancer cells [126].

The involvement of A_3_ARs in diabetic neuropathy was also investigated. It has been demonstrated that IB-MECA treatment ameliorates mechanical hyperalgesia and thermal hypoalgesia in STZ-treated mice. Moreover, reduced expression or functionality of A_3_ARs promoted diabetic neuropathy development [127].

It is well established that long-lasting treatments with opioids lead to hyperalgesia and tolerance to drugs, reducing the analgesic effect of opioids in chronic pain [128,129]. In a rodent model, it has been reported that the opioid adverse effects seem to be linked to reduced A_3_ARs signaling. In fact, the stimulation with A_3_AR agonists ameliorates pain sensitivity suggesting that selective A_3_AR agonists may be used in addition to opioids for chronic pain management [130]. Importantly, it has been reported that the antinociceptive effects of A_3_AR agonists persist at least up to 2 weeks of treatment, suggesting that stimulation of A_3_ARs does not induce tolerance [87]. 

A recent study reports the effect of a new A_3_AR agonist, AL170, in a rat model of colitis. AL170 mitigates the colonic damage and inflammation, reducing the release of TNF-α, IL-1β, and myeloperoxidase. AL170 was demonstrated to have an efficacy comparable to that of dexamethasone, one of the most used drugs in the colitis treatment and other inflammatory bowel diseases [131]. The activation of A_3_ARs resulted able to decrease the infiltration of inflammatory cells and the production of inflammatory mediators thus softening visceral pain [131]. A further study revealed that the treatment with A_3_AR agonists is useful in another model of abdominal pain induced by colitis. In this model, Cl-IB-MECA and MRS5980 decreased visceral hypersensitivity in the postinflammatory phase as well as in the and persistence one and showed effectiveness comparable to that of linaclotide, a drug used for the treatment of irritable bowel syndrome [132,133].

The possibility to exploit A_3_AR agonists in rheumatic pathologies has been studied, starting from the observation that A_3_ARs are up-regulated in synovial tissue and peripheral blood mononuclear cells in rheumatoid arthritis patients. Treatment with A_3_AR agonists leads to an improvement of symptoms and clinical signs [90,93,113]. Another potential therapeutic approach for arthritis could be represented by allosteric enhancer, in order to exploit the anti-inflammatory action of A_3_ARs and the high adenosine concentrations typical of inflammatory pathologies. LUF6000, an A_3_AR allosteric modulator, was showed to reduce inflammation in models of adjuvant- and monoiodoacetate-induced arthritis [134]. Even if the analysis of the nociceptive activity was not comprised in these studies, it is reasonable to hypothesize that a decreased inflammation would be accompanied by a reduction in pain.

**Table 4:** A_3_AR ligands with antinociceptive effects in preclinical models of pain.

Ligand	Pharmacological Behavior	Pain Model	Species	Route of Administration
AR170	A_3_AR agonist	colitis-induced visceral hypersensitivity	rat	i.p. [131]
Cl-IB-MECA	A_3_AR agonist	chemotherapy-induced mechanical allodynia	mouse	i.p. [115]
		CCI-induced mechanical allodynia	mouse	i.p. [115]
		bone cancer-induced mechanical allodynia	rat	i.p. [126]
		colitis-induced visceral hypersensitivity	rat	i.p. [133]
IB-MECA	A_3_AR agonist	chemotherapy-induced mechanical allodynia	mouse/rat	i.p. [115,122]
		CCI-induced mechanical allodynia	mouse	i.p. [115]; i.t. [118]
		STZ-induced mechanical allodynia and thermal hyperalgesia	mouse	i.p. [127]
		opioid-induced thermal hyperalgesia	rat	p.o. [130]
		tibial nerve injury-induced mechanical allodynia	rat	i.p. [120]
MRS1898	A_3_AR agonist	chemotherapy-induced mechanical allodynia	mouse	i.p. [115]
		CCI-induced mechanical allodynia	mouse	i.p. [115]
MRS5698	A_3_AR agonist	CCI-induced mechanical allodynia	rat	s.c., i.p., i.v. [117]; i.t. [118]
		spared nerve injury-induced mechanical allodynia	rat	s.c., i.p., i.v. [117]
		SNL-induced mechanical allodynia	rat	s.c., i.p., i.v. [117]
		bone cancer-induced mechanical allodynia	rat	s.c., i.p., i.v. [117]
		chemotherapy-induced mechanical allodynia	rat	s.c., i.p., i.v. [117]; i.t. [124]
		opioid-induced thermal hyperalgesia	rat	p.o. [130]
MRS5980	A_3_AR agonist	colitis-induced visceral hypersensitivity	rat	i.p. [133]
MRS7422	A_3_AR agonist	CCI-induced mechanical allodynia	mouse	p.o. [121]

i.p. (intraperitoneal); CCI (chronic constriction injury); i.t. (intrathecal); STZ (streptozotocin); p.o. (per os); s.c. (subcutaneous); i.v. (intravenous); SNL (spinal nerve ligation).

## 3. Conclusions

The modulation of ARs, especially their activation, induces potent antinociceptive effects in diverse preclinical models of acute and chronic pain. Nevertheless, the efficacy of AR ligands for the pharmacological treatment of pain in humans is still ambiguous and it has also yet to be determined whether ARs modulation could be exploited to inhibit spontaneous pain. Many hopes were initially placed on A_1_AR agonists, but cardiovascular side effects prevented their progress in the clinic, at least with regard to their systemic administration. To get around these obstacles, alternative strategies have been proposed to continue exploiting the huge potential of adenosine and its receptors in pain management. Partial agonist and allosteric modulators of ARs have been tested in preclinical settings revealing potent antinociceptive effects with fewer side effects than conventional full agonists. Furthermore, localized activation of ARs has been proposed as a valid alternative to systemic delivery to maintain efficacy and reduce side effects, especially considering the ubiquitous localization of ARs in the human body. Exogenous ectonucleotidases as well as metabolizing enzyme inhibitors could increase the extracellular concentration of the short-living mediator adenosine, enhancing its nociceptive effects. As reviewed here, these alternative pharmacological approaches have shown promising results in preclinical models of pain and could offer a means to overcome the issues encountered so far by AR ligands in the clinic. Overall, the data summarized in this review highlight the therapeutic potential of ARs as pharmacological targets for the treatment of acute and chronic pain and the need to develop new and more effective strategies to exploit this potential. 

## Abbreviations

GPCR	G protein coupled receptors
AR	adenosine receptor
AC	adenylate cyclase
cAMP	cyclic adenosine monophosphate
ATP	adenosine triphosphate
ADA	adenosine deaminase
PKA	protein kinase A
PLC	protein lipase C
IP3	inositol triphosphate
DAG	diacylglycerol
ERK	extracellular signal-regulated kinase
NO	nitric oxide
cGMP	cyclic guanosine monophosphate
PKG	protein kinase G
PAG	periaqueductal gray
CCI	chronic constriction injury
CFA	complete Freund’s adjuvant
SCNL	sciatic nerve ligation
KO	knockout
PAP	prostatic acid phosphatase
SCI	spinal cord injury
SNL	spinal nerve ligation
STZ	streptozotocin
ROS	reactive oxygen species
NF-κB	nuclear factor kappa-light-chain-enhancer of activated B cells
TNF-α	tumor necrosis factor-α
iNOS	inducible nitric oxide synthase
SIN	sciatic inflammatory neuropathy
PKC	protein kinase C
IL	interleukin
PI3K	phosphoinositide 3-kinase
MAPK	mitogen-activated protein kinase
CREB	cAMP response element-binding protein
